# Neglected Infections of Poverty among the Indigenous Peoples of the Arctic

**DOI:** 10.1371/journal.pntd.0000606

**Published:** 2010-01-26

**Authors:** Peter J. Hotez

**Affiliations:** Sabin Vaccine Institute and Department of Microbiology, Immunology, and Tropical Medicine, The George Washington University, Washington, D.C., United States of America

The neglected tropical diseases are not always exclusively tropical as defined by their endemicity between the Tropic of Cancer in the northern hemisphere and in the Tropic of Capricorn in the southern hemisphere. Indeed, in previous articles, it has been pointed out that neglected infections occur wherever extreme poverty occurs [1], even in pockets of poverty in North America and Europe [2]–[4]. One of the more dramatic illustrations of poverty as the single most important determinant of neglected infections among human populations is the observation that these conditions occur among the poorest people living in the Arctic region [1]. The actual definition of Arctic region varies (Figure 1), with some experts basing it on the land and sea north of the Arctic Circle (66° 33′ N), while others, the area north of the 10°C (50°F) July isotherm corresponding to the tree line [5]. There are seven countries with significant territory in the Arctic, including Canada, Finland, Greenland, Norway, Russia, Sweden, and the United States (Alaska only) [6], although Iceland is also sometimes included. Approximately two million people live north of the Arctic Circle, with 60% living in Arctic Russia [6]. A high percentage of these populations represent aboriginal or indigenous peoples, terms still not well defined because such groups are so often marginalized that there is inadequate data for their numbers and socioeconomic parameters [7]. Arctic aboriginal peoples represent only a small percentage of the estimated 370 million indigenous people globally [7].

**Figure 1:**
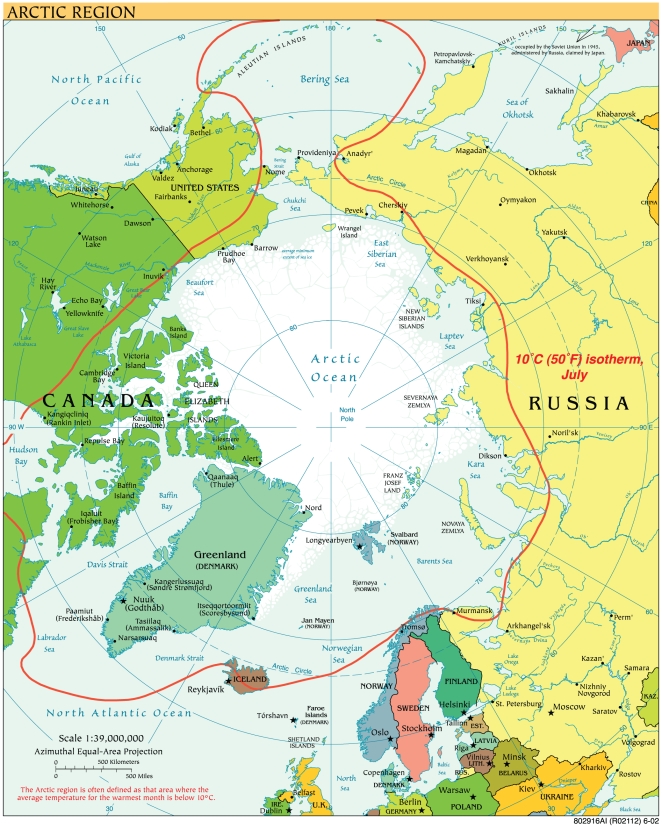
Map of the Arctic Region. From Wikimedia (http://commons.wikimedia.org/wiki/File:Arctic_big.svg), accessed August 22, 2009.

## Indigenous Populations in the Arctic

In Canada, the major indigenous peoples of the Arctic are the roughly 50,000–60,000 Inuit (Table 1) [8],[9]. This population represents the smallest of Canada's three major aboriginal populations, which also include one million people belonging to more than 600 First Nations peoples, and 300,000 Métis [9]. Approximately one-half of the Inuit live in the northeastern territory of Nunavut (where they constitute 85% of the population), less than one-fourth in northern Quebec (i.e., Nunavik), and the remainder in northern Labrador or in Yukon and the Northwest Territories [10]. Many of the Inuit communities are extremely isolated and accessible only by airplane or boat [10]. Another 50,000 Kalaalit Inuit make up almost 90% of the population of Greenland [10]. The Kalaalit Inuit are considered closely related to the Inuit in northern Canada [9]. In Alaska there are roughly 100,000 indigenous people (known as Alaskan natives) that make up 16% of the state's population [10]. In addition to the Inuit Inupiat, the Alaskan natives also include the Aleut and Alutiiq peoples who separated from the Inuit approximately 4,000 years ago, and are differentiated in part by their historical contacts with indigenous peoples living in Siberia, and with Native Americans living on the Northwest Coast of the United States (including the Athabascan) [10]. In Siberia, according to the Russian Association of Indigenous Peoples of the North, there is a total aboriginal population of 250,000 from more than 40 ethnic groups, including approximately 40,000 Nenets, 35,000 Evenk, 30,000 Khanty, and 20,000 Even [11]. Finally, in the Nordic countries of Norway, Finland, and Sweden live roughly 60,000 Sami [12], although the exact number is considered by some controversial because of their high degree of assimilation within the Nordic populations.

**Table 1:** Major indigenous populations living in the Arctic.

Indigenous Group	Major Country or Area	Estimated Population [Reference]
Inuit	Canadian Arctic	50,485 in 2006 [8]; 60,000 in 2009 [9]
Kalaalit Inuit	Greenland	50,000 in 2007 [10]
Alaska natives: Aleut, Alutiiq, Inupiak-Eskimo, others	Alaskan Arctic	107,000 in 2006 [10]
Russian-Siberian natives (groups ≥20,000): Nenets, Evenk, Khanty, Even,	Russian-Siberian Arctic	250,000 [11]
Sami	Nordic countries	60,000 [12]

A review of literature (papers or abstracts in English only), using as MEDLINE/PubMed medical subject headings (MeSHs) the specific diseases listed as the neglected tropical diseases on the *PLoS Neglected Tropical Disease* Web site (http://www.plosntds.org) and the geographic regions and countries of the Arctic, over the last two decades but with emphasis on the last 10 years (reference lists of identified articles and reviews were also manually searched), reveals a modest literature on infectious diseases of the North American aboriginal populations but far less on the larger Russian-Siberian populations. This observation likely reflects the bias introduced by examining only literature in English, together with more than 20 years of outstanding work by several Canadian universities and institutes, particularly studies in Nunavik conducted by investigators at McGill University and in Alaska by the Arctic Investigations Program of the United States Centers for Disease Control and Prevention.

## Arctic Infectious Diseases and Other Health Disparities

In their recent overview on indigenous health Gracey and King point out that globally, the almost 400 million indigenous people live in poor health linked to “poverty, malnutrition, overcrowding, poor hygiene, environmental contamination, and prevalent infections” [7]. Indeed, compared to non-aboriginal Canadians the Inuit suffer disproportionately from high rates of chronic conditions such as smoking and obesity, and have a life expectancy that is roughly 8–12 years shorter [10]. According to the Library of the Canadian Parliament, the Inuit exhibit high rates of injuries from accidents, and their suicide rates are 11 times the national average [8]. In addition, the Inuit living in Canada and Greenland suffer from high incidence of alcohol and other substance abuse, sexual abuse, and family violence, and often live in overcrowded conditions [8],[13]. They live in isolated communities and their employment is frequently limited to either traditional activities of hunting and fishing, or working as wage-earners in the Government or service industries [8]. Unemployment rates in Canada are high [8], and a 2001 survey by the Canadian Government revealed that 34% of Inuit living in northern Canada reported that at certain times of the year their drinking water is contaminated [9].

It is not surprising that in this setting of stress, socioeconomic deprivation, and environmental degradation infectious diseases would flourish. Indeed, overall the world's indigenous people in general suffer from high rates of infections such as ectoparasitic skin infestations, upper and lower respiratory tract infections and central nervous system infections from bacterial invasive organisms and tuberculosis, childhood illnesses (due to low vaccine coverage), diarrheal and intestinal helminth infections, urinary tract infections, bone and musculoskeletal infections, and in some cases, HIV/AIDS and malaria [7]. Shown in Table 2 are listed some of the major infections found with increased frequency (relative to their nonaboriginal counterparts) among aboriginal populations living in Canada, Greenland, and Alaska. Higher rates of respiratory infections caused by *Haemophilus influenzae* type b, *Streptococcus pneumoniae*, and *Bordetella pertussis* have been noted among Alaska natives [14], and other invasive bacterial infections among the Inuit in Greenland [15]. With increasing vaccine coverage for *H. influenzae* type b, a new serotype, *H. influenzae* type a, has emerged among children less than 2 years of age among Alaskan natives and the Inuit in Canada [16]. Both otitis media and lower respiratory tract infection rates are higher among Inuit children in northern Quebec compared to non-native populations, with hospital admission rates for lower respiratory tract infections up to 10 times higher [17]. Among the risk factors for hospital admissions for Inuit children less than 2 years of age with lower respiratory tract infections (especially respiratory syncytial virus infection) are smoking in pregnancy, overcrowding, and non-breast feeding [18]. While the risk of hepatitis A in Canada is considered low [19], both hepatitis B and C are important causes of chronic liver disease among Alaskan Native people [20]. However, among the Inuit in Greenland a special variant of hepatitis B may be present that produces mild or dormant disease [21]. Hepatitis E infection is prevalent among the Canadian Inuit and there is interest in determining whether caribou meat may serve as a source [22]. Gastrointestinal infection with *Helicobacter pylori* among the Inuit and Alaskan natives has been observed [10]. High rates of iron deficiency anemia have been described in Arctic populations infected with *H. pylori* [10], [23]–[25], which presumably occurs through gastritis and gastrointestinal bleeding.

**Table 2:** Selected infectious diseases including neglected infections of poverty among Arctic indigenous populations.

Disease Group	Infectious Disease	Status (Occurrence) or Comment	Reference
**Respiratory and other invasive diseases**	Bacterial respiratory infections caused by *Haemophilus influenzae* type b, *Streptococcus pneumoniae*, and pertussis	Alaskan native children	[14]
	Bacterial invasive diseases caused by *S. pneumoniae*, *Escherichia coli*, *Staphylococcus aureus*, and other Staphylococci	Greenland Inuit	[15]
	Bacterial invasive diseases (meningitis, pneumonia, septic arthritis) caused by *H. influenzae* type a	Overall incidence 0.9 per 100,000; among indigenous children<2 y.o.; incidence of 21 per 100,000 in Alaska, 102 per 100,000 in northern Canada	[16]
	Acute otitis media and lower respiratory tract infection	Admission for lower respiratory tract infection 10 times higher in northern Quebec (Nunavik) compared to other Canadian populations	[17]
	Respiratory syncytial virus and other respiratory tract infections	Risk factors in Canadian arctic among children<2 y.o. include smoking in pregnancy, overcrowding, Inuit race, and non-breast feeding	[18]
**Hepatitis**	Hepatitis A	Low risk in Canada	[19]
	Hepatitis B and C	Important cause of liver failure among Alaska natives, although Hepatitis B produces a more benign disease in Greenland Inuit	[20],[21]
	Hepatitis E	3% seroprevalence among Canadian Inuit	[22]
	*Helicobacter pylori* infection	Common in Canadian Inuit and Alaska native populations; linked to iron deficiency anemia	[23]–[25]
**Neglected infections of poverty**	Trichinellosis	Transmitted from polar bear and walrus. Incidence rates of 11 per 100,000 per year in northern Canada; 1.8 per 100,000 in Alaska, compared to 0.05–0.06 per 100,000 in the United States and Canada overall	[26]–[29]
	*Trichinella nativa* infection		
	Diphyllobothriasis	Sporadic cases in the Arctic region	[30]–[32]
	*Diphyllobothrium* spp. infection (		
	Cystic echinococcosis	Incidence rates highest in northern Russia-Siberia where sled and herding dogs are widely used	[33]
	*Echinococcus granulosus* infection		
	Alveolar echinococcosis	Sporadic cases in Western Alaska; more prevalent in Russia	[34]
	*Echinococcus multilocularis* infection		
	Toxoplasmosis	60% seroprevalence in Nunavik; association with caribou and seal consumption, and contaminated drinking water	[36],[37]
	*Toxoplasma gondii* infection		
	Giardiasis	Possible role for zoonotic transmission from seals	[38],[39]
	*Giardia* spp. infection		

## Arctic Neglected Infections of Poverty

Parasitic helminth and protozoan infections are also common among the Inuit in Canada and Greenland and among Alaskan natives. The concept of neglected infections of poverty among the Inuit and Alaska natives was introduced previously [1], and this concept is expanded here. Many of these parasitic infections are foodborne and are transmitted through uncooked or inadequately prepared meats from polar bear and sea mammals such as walrus or seals, while others are zoonoses transmitted from close associations with the livestock unique to the Arctic region such as reindeer and elk.

### 

#### Helminth infections

The major helminthiases among indigenous Arctic populations are trichinellosis, diphyllobothriasis, and echinococcosis. Arctic outbreaks of trichinellosis have been described since the 1940s when a large outbreak in West Greenland from infected walrus and polar bear resulted in more than 400 cases and 37 deaths [26]. Ultimately, more an estimated 40,000 Greenland Inuit were noted to be infected by the mid-1980s [27].

More recently, outbreaks of trichinellosis were noted among the Canadian Inuit [27],[28]; with the largest involving 42 individuals living at the northern tip of Nunavik [27],[28]. MacLean and his colleagues (see In Memoriam, below) reviewed the incidence rates of Arctic trichinellosis and reported that in the United States, Alaska had the highest rate, with 1.8 cases per 100,000 per year, compared with 0.05 cases per 100,000 per year for the entire country, while in the northernmost region of Canada the incidence rate was as high as 11 cases per 100,000 per year, compared with 0.06 cases per 100,000 per year for all of Canada [27]. The natural history and etiology of Arctic trichinellosis is quite distinct from other forms of this helminth infection. In addition to its relationship to consumption of polar bear and walrus meat, the Arctic infection is caused by a unique *Trichinella nativa* species that has the ability to withstand freezing temperatures [27],[28]. This feature presumably allows the nurse cell stages of the parasite to survive in the flesh of carcasses deposited on the ice long enough until they are ingested by another mammal. In humans *T. nativa* infection also exhibits several unique clinical features. Whereas primary infection resembles other forms of trichinellosis, i.e., a short intestinal phase followed by the classical features associated with muscle invasion including fever, muscle pain and weakness, and edema, recurrent infection with *T. nativa* produces largely prolonged diarrhea [27],[28]. Both forms of the illness are associated with eosinophilia [27],[28]. In Nunavik, based on the observation that 11 outbreaks of trichinellosis occurred between 1982 and 1999 with all but two caused by ingesting walrus meat, an innovative prevention program has been launched in which regional laboratories test the meat of harvested walrus for the presence of *Trichinella* larvae and then rapidly disseminate the testing results to potentially affected communities [29]. A key feature of the program was the participation and willingness of the hunters to provide meat samples for testing and then wait for test results before distributing or consuming the meat.

In addition to trichinellosis, diphyllobothriasis (fish tapeworm) is endemic to Alaska and elsewhere in the Arctic region [30]–[32], and both cystic and alveolar forms of echinococcosis still occur. Rausch [33] has extensively studied the northern biotype of *Echinococcus granulosus*, the etiologic agent of cystic echinococcosis in the Arctic region where natural enzootic life cycles between wolf and wild reindeer and wolf and elk exist. At one time human cystic echinococcosis was highly prevalent among aboriginal populations living in Alaska and the Canadian Arctic as a consequence of using dogs for hunting and herding activities [33]. However, as dogs have been replaced by mechanized vehicles in the region, the rates of human cystic echinococcosis have declined precipitously in North America, although the rates of infection remain high and may even be increasing in northern Russia and Siberia [33]. In North America, alveolar echinococcosis caused by *Echinococcus multilocularis* is a rare infection, although sporadic cases have been described in a small hyperendemic area in Western Alaska [34]. The infection is believed to still be prevalent in Russia [34]. A more exhaustive listing of the helminth and other parasites in the Arctic is found in reference [35].

#### Protozoan infections

In terms of prevalence and disease burden toxoplasmosis is probably the most important parasitic infection in the North American Arctic [36],[37]. In 1987 an outbreak of toxoplasmosis was investigated in Nunavik after physicians working in a regional hospital noted that four of thirty women seroconverted during their pregnancies [36]. Seroconversion was linked to skinning of animals and consumption of caribou meat and seal liver and dried seal meat [36], and it was presumed that transmission resulted from ingestion of bradyzoites from either caribou or seal, or both. In a follow-up cross-sectional study among 917 adult Inuit in Nunavik the overall seroprevalence for *Toxoplasma gondii* infection was considered very high at 60% [37]. A somewhat surprising association was a link observed between *T. gondii* infection and consumption of unfiltered surface water. Given the overall absence of cats in this area there is interest in determining whether lynx (a wild cat in the region) could be responsible, and whether infective oocysts could wash into the sea by surface water runoff thereby contaminating the marine environment [37]. Giardiasis and cryptosporidiosis also occur in the Arctic region [38],[39]. The finding of *Giardia* parasites in seals in Nunavik suggests the possibility that this infection is acquired through zoonotic transmission [39].

## Concluding Statement

The high rates of neglected infection of poverty among indigenous populations living in Alaska, Greenland, and the Canadian Arctic require further investigation, especially the identification and role of definitive and intermediate hosts in the transmission of toxoplasmosis, trichinellosis, and giardiasis. Given the high rates of toxoplasmosis a program of newborn screening should be implemented for these populations, so that at-risk infants could begin antiprotozoan chemotherapy. The dearth of information on neglected infections among indigenous populations living in Russia and Siberia also suggests that increased efforts are required for active surveillance for the most common infections in this region and to step up measures for studying the area's unique ecology and infection transmission dynamics. Ultimately, programs for prevention of neglected infections may need implementation for all of the indigenous peoples living in the Arctic region.

## IN MEMORIAM: John Dick Fleming MacLean, MD, FRCP(C), MRCP (UK), DCMT (London), 1940–2009

**Figure 2:**
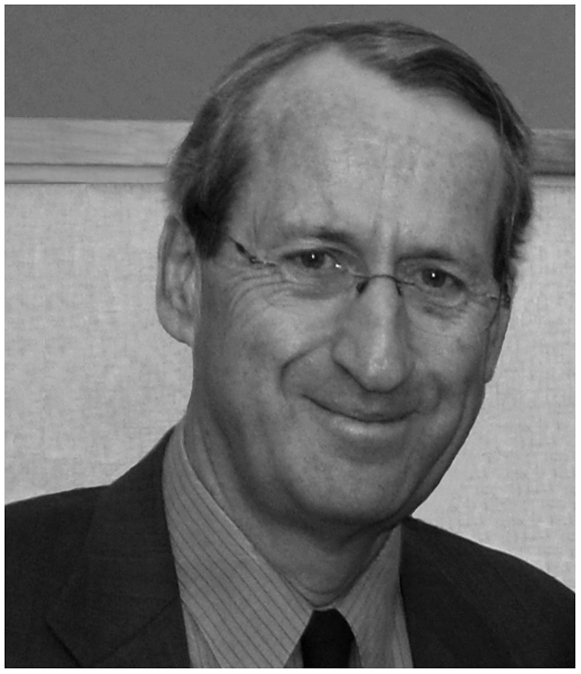
John Dick Fleming MacLean, MD, FRCP(C), MRCP (UK), DCMT (London), 1940–2009.

Dr. J. Dick MacLean (Figure 2) completed medical training at Queens University in 1966. He subsequently specialized in Internal Medicine and Tropical Medicine (London School of Hygiene & Tropical Medicine). He then taught at the Gombak Aborigine Hospital in Malaysia, the University of Nairobi in Kenya, and the University of Hawaii Program in Japan before returning to McGill University in 1980. He was quickly named director of the McGill University Centre for Tropical Diseases (TDC) which he nurtured and defended for almost three decades. Under his stewardship, the TDC grew into a renowned national referral center committed to patient care, both clinical and basic research, and especially training and education. Countless students, colleagues, and organizations have benefited from his knowledge of clinical tropical medicine over the years. Dr. MacLean gave generously of his time and expertise, including to the Public Health Agency of Canada through service on expert committees (e.g., Canadian Committee to Advise on Tropical Medicine and Travel), Canadian and American International Health Societies (e.g., Canadian Society for International Health, Canadian University Consortium for Health in Development, American Society of Tropical Medicine and Hygiene) and many others. He published 92 papers in the peer-reviewed literature and contributed many chapters to texts focused on tropical medicine. Perhaps paradoxically for a “tropical diseases doctor,” in Canada his name will always be linked to research on new parasitic diseases in northern communities (e.g., endemic trichinosis, *Metorchis conjunctus*). Dr. MacLean died on 22 January 2009 following complications from surgery. In recognition of his exemplary career, McGill University has re-named the TDC the J. D. MacLean Centre for Tropical Diseases. His greatest legacy, however, is reflected in the generations of students, trainees, and colleagues who benefited from his teaching and counsel and who were inspired by him to become better citizens of the world.

*Contributed by Theresa W. Gyorkos, Michael Libman, and Brian J. Ward*
